# Fabrication of a Novel Z-Scheme AgBiO_3_/BiOCl Heterojunction with Excellent Photocatalytic Performance towards Organic Pollutant

**DOI:** 10.3390/ma17184615

**Published:** 2024-09-20

**Authors:** Shuai Fu, Zhiquan Huang, Yanhong Wang, Bingqian Zheng, Wei Yuan, Leicheng Li, Peiyuan Deng, Huijie Zhu, Hui Zhang, Bo Liu

**Affiliations:** 1 Henan International Joint Laboratory of New Civil Engineering Structure, College of Civil Engineering, Luoyang Institute of Science and Technology, Luoyang 471023, China; 2 Henan Engineering Research Center of Water Quality Safety in the Middle-Lower Yellow River, Henan Green Technology Innovation Demonstration Base, Luoyang 471023, China; 3 School of Ecology and Environment, Zhengzhou University, Zhengzhou 450001, China; 4 School of Environmental and Municipal Engineering, North China University of Water Resources and Electric Power, Zhengzhou 450046, China; 5 Biological Species Resource Research Key Laboratory, Zhengzhou Normal University, Zhengzhou 450044, China; 6 Laboratory of Functional Molecular and Materials, School of Physics and Optoelectronic Engineering, Shandong University of Technology, Zibo 255000, China

**Keywords:** AgBiO_3_/BiOCl, Z-scheme, heterojunction, photo-degradation, ciprofloxacin

## Abstract

A novel and highly efficient photocatalyst of a AgBiO_3_/BiOCl heterojunction has been developed via a facile water bath and in situ precipitation method. The photocatalytic activities of the catalysts were investigated by the degradation of ciprofloxacin (CIP) under visible-light irradiation (>420 nm). The experiment results revealed that the photocatalytic performance of the optimized AgBiO_3_/BiOCl heterojunction was much higher than pure AgBiO_3_ and BiOCl. The degradation efficiency of the as-prepared AgBiO_3_/BiOCl heterojunction (ABC-30) for CIP could reach 88% within 160 min, with 2.89 and 3.76 times higher activity than pure AgBiO_3_ and BiOCl, respectively. The improved photocatalytic performance of AgBiO_3_/BiOCl was attributed to the synergistic effect of the enhanced light absorption range and effective separation and transfer of the photo-induced charge carrier. The optimized heterojunction showed broad-spectrum catalytic activities towards various organic contaminants. The degradation efficiencies varied with the nature of the pollutant and decreased in the following order: Lanasol Red 5B (100%) > methyl orange (99%) > methylene blue (98%) > tetracycline (92%) > ciprofloxacin (88%) > ofloxacin (85%) > norfloxacin (78%) > rhodamine B (59%) > metronidazole (43%) > phenol (40%) > carbamazepine (20%). Furthermore, the trapping experiments and ESR indicated that superoxide radicals and holes were the main reactive species.

## 1. Introduction

Ciprofloxacin is an antibacterial drug, which has been widely used domestically and internationally in recent years [1,2]. Nevertheless, the discharge of pharmaceutical wastewater in China has rapidly increased with the widespread application of antibiotics [3,4]. The wastewater contained high concentrations of organic matter, making the antibacterial ability of the wastewater stronger and the biological toxicity greater, which caused it to be difficult to biodegrade. This would lead to environmental pollution such as water bodies, soil, and groundwater [5,6]. Generally speaking, the common traditional methods for removing pollutants from water mainly include coagulation [7], membrane separation [8], biochemistry [9], and activated carbon adsorption [10]. However, it has been found that CIP degradation by traditional methods is poor, which often not only failed to achieve ideal degradation efficiency, but if they were not operated properly, they were also highly likely to produce other ecological toxicity [11,12]. Current research has found that photocatalytic technology was an efficient and environmentally companionable technology that could be extensively applied in sewage treatment fields, which was also considered to have great prospects in the field of organic pollutant degradation [13,14,15,16,17].

Bismuth oxychloride (BiOCl) was an emerging type of semiconductor compound. Its excellent photocatalytic activities came from an open layered structure and indirect band gap orbital transition form, which reduced the recombination of photo-generated holes and electrons [18,19,20]. Moreover, due to the difference in electronegativity between the elements, the open layered crystal structure of BiOCl could generate sufficient space to polarize atoms and orbitals, allowing for the effective separation and transfer of a photo-induced charge carrier [21,22]. However, the applications of BiOCl were confined due to several inherent drawbacks, comprising low solar energy utilization and rapid recombination of the photo-induced charge carrier.

Among numerous modification methods, the construction of a heterojunction has been proven to be an effective approach to elevate photocatalytic activity. The most common way to construct heterojunctions was to assemble two different semiconductor materials that possess unequal band gaps in a band-aligned manner [23,24,25]. Thereby, the potential difference was utilized to enhance the separation and transfer of the photo-induced charge carrier and expand its light absorption response range. Importantly, silver bismuth oxide (AgBiO_3_) was an almost black crystalline material with a narrow band gap width [26,27]. As a new type of catalyst, it had the characteristics of high stability, high visible-light utilization, and strong redox properties, and was considered an excellent semiconductor material. Compared with traditional catalysts, it had better photocatalytic activity in removing organic pollutants in water. For example, Zhao prepared a AgBiO_3_/g-C_3_N_4_ heterojunction via the ion-exchange method and calcination method; 50 wt.% AgBiO_3_ in AgBiO_3_/g-C_3_N_4_ showed an excellent photocatalytic performance towards BPA, reaching 95.75% [28]. Dutta synthesized a novel CNTs@CuBi_2_O_4_/AgBiO_3_ to explore the visible-light photocatalytic performance towards MB, which showed a supreme photocatalytic efficiency of 97.94%. Moreover, after ten cycles, the photocatalytic activity remained 89.50%, proving its high stability [29].

This paper employed a simple, mild, and user-friendly method to synthesize a AgBiO_3_/BiOCl heterojunction. Flake-like BiOCl was prepared by the water bath method, and the AgBiO_3_/BiOCl heterojunction was prepared by the in situ precipitation method. The ciprofloxacin was used to evaluate the photocatalytic activity under visible-light irradiation. In addition, the effects of the photocatalytic process such as the CIP concentration, pH, and ions were also studied.

## 2. Materials and Methods

### 2.1. Chemicals

Bismuth nitrate pentahydrate (Bi(NO_3_)_3_·5H_2_O), silver nitrate (AgNO_3_), sodium chloride (NaCl), sodium bismuthate (NaBiO_3_), and ethylene glycol (CH_2_OH)_2_ were purchased from Sinopharm Chemical Regent (Shanghai, China). All chemical reagents were of analytical reagent grade.

### 2.2. Synthesis of BiOCl Nanosheet

The BiOCl with oxygen vacancies was prepared via a simple water bath method [24,30]. Typically, 1.5 mmol Bi(NO_3_)_3_·5H_2_O was dissolved in 30 mL ethylene glycol, marking solution A. Then, 3 mmol NaCl was dissolved in 20 mL deionized water, which was added dropwise into solution A. The mixed solution was stirred magnetically for 3 h at 90 °C under water bath conditions. The final product was centrifuged with deionized water and ethanol several times, and then dried at 60 °C for 24 h.

### 2.3. Synthesis of AgBiO_3_/BiOCl Heterojunction

Firstly, 0.3 g of BiOCl was added into 60 mL of deionized water, and sonicated for 20 min. Then, 0.06 g AgNO_3_ was subsequently added. After 30 min additional sonication, 0.12 g NaBiO_3_ was added to the above solution and stirred for 30 min at room temperature. Subsequently, the mixed solution was centrifuged at 6000 rpm. Eventually, the final product was washed with deionized water and ethanol several times, and then dried at 80 °C for 24 h. The mass ratios of AgBiO_3_ and BiOCl were 1%, 10%, 30%, 50%, and 70%, which are referred to as ABC-1, ABC-10, ABC-30, ABC-50, and ABC-70. Pristine AgBiO_3_ was prepared via similar procedures without adding BiOCl.

### 2.4. Characterization

The crystal phases of the as-prepared samples were characterized using a Bruker-D8-Axs diffractometer system with Cu Kα radiation (λ = 0.15406 Å). The morphological details of the samples were investigated with TEM (JEM-2100) and SEM (SU8010). The electron spin resonance (ESR) spectra were received on a Bruker ER300-SRC instrument. The 5,5-dimethyl-l-pyrroline-N-oxide (DMPO) was used to trap the ESR spectra by mixing 0.1 g of the photocatalyst in 30 mM DMPO solution with a 45 mL aqueous dispersion for DMPO-•OH.

### 2.5. Evaluation of Visible-Light-Driven Photocatalytic Performance

The photocatalytic performance of the as-synthetized catalysts was tested by the degradation of CIP under visible-light irradiation (350 W Xe lamp with a UV cutoff filter (Nanjing Xujiang Electromechanical Factory, Nanjing, China), λ > 420 nm). Typically, 40 mL ciprofloxacin solution (rhodamine B, methyl orange, methylene blue, tetracycline hydrochloride, Lanasol Red 5B, phenol, and metronidazole, 40 mg/L) containing 40 mg catalysts was stirred for 30 min in the dark to reach adsorption–desorption equilibrium. Subsequently, 3 mL solution was withdrawn at a scheduled interval. The concentrations of CIP in the supernatant were measured with a UV–vis spectrophotometer (UV-2450). The degradation efficiency was calculated by the value of (*C*_0_ − *C_t_*)/*C*_0,_ where *C*_0_ was the initial concentration and *C_t_* was the instant concentration of the pollutant.

## 3. Results

### 3.1. Physicochemical Properties

**Figure 1:**
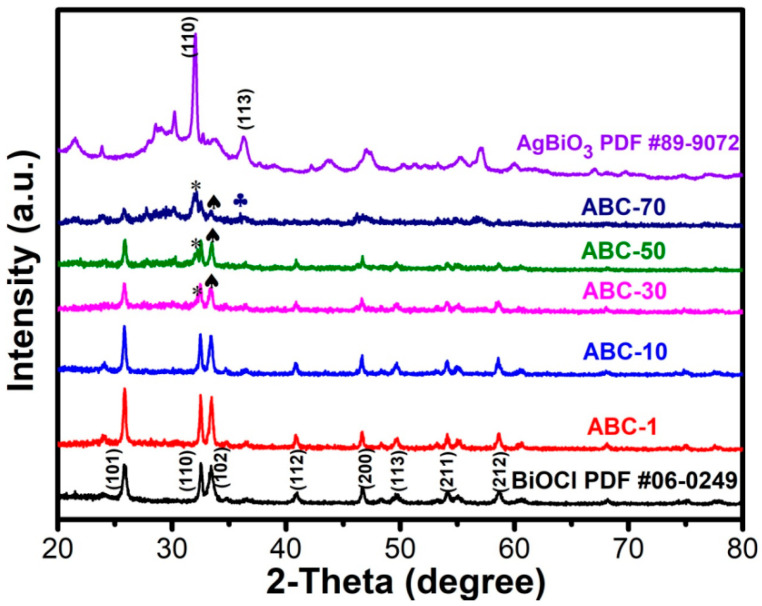
XRD patterns of the standard diffraction card of AgBiO_3_, BiOCl, the AgBiO_3_/BiOCl heterojunctions.

The typical diffraction patterns of the as-synthetized samples are shown in Figure 1. They reveal that the pristine BiOCl possessed the main characteristic diffraction peaks at 25.8°, 32.5°, 33.4°, 40.8°, 46.6°, 49.7°, 54.1°, and 58.6°, corresponding to the (101), (110), (102), (112), (200), (113), (211), and (212) crystal planes of the tetragonal BiOCl (JCPDS NO: 06-0249), respectively. The primary diffraction peaks for the pristine AgBiO_3_ at 31.7° and 35.9° were assigned to the (110) and (113) crystal planes, respectively, which were in accordance with the standard diffraction patterns of AgBiO_3_ (JCPDS 89-9072). When the AgBiO_3_ was loaded on the BiOCl surface, the XRD pattern of ABC-1 and ABC-10 did not exhibit obvious differences compared to the pristine BiOCl, ascribed to the low content of AgBiO_3_. As the amounts of AgBiO_3_ increased, the diffraction peaks at 2θ = 31.7° and 35.9°, corresponding to the (110) and (113) plane of AgBiO_3_, gradually appeared in the AgBiO_3_/BiOCl heterojunctions. No impurity peaks in the spectrum indicated a high purity of the catalysts.

**Figure 2:**
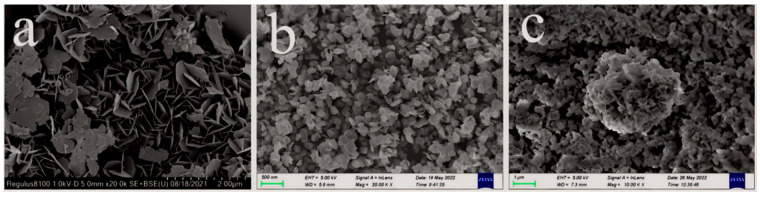
FE-SEM of (**a**) AgBiO_3_, (**b**) BiOCl, and (**c**) ABC-30 heterojunction.

FE-SEM was used to investigate the morphology and structure of the AgBiO_3_, BiOCl, and ABC-30 heterojunction. As shown in Figure 2a, the pristine AgBiO_3_ revealed a flower-like structure composed of nanosheets with diameters around 500 nm. As shown in Figure 2b, the pristine BiOCl consisted of smooth nanosheets with a diameter of 250–500 nm. For the AgBiO_3_/BiOCl heterojunction, the flower-like AgBiO_3_ was closely contacted with the surface of the BiOCl (Figure 2c), suggesting excellent photocatalytic activity.

**Figure 3:**
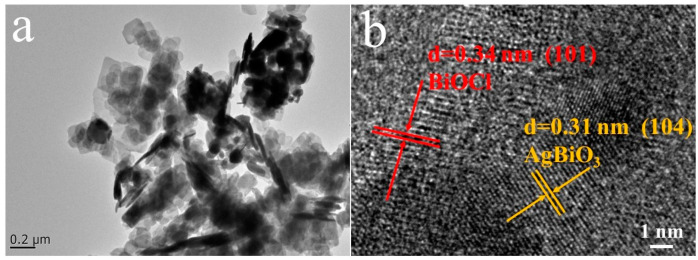
(**a**) The TEM and (**b**) HRTEM image of ABC-30 heterojunction.

**Figure 4:**
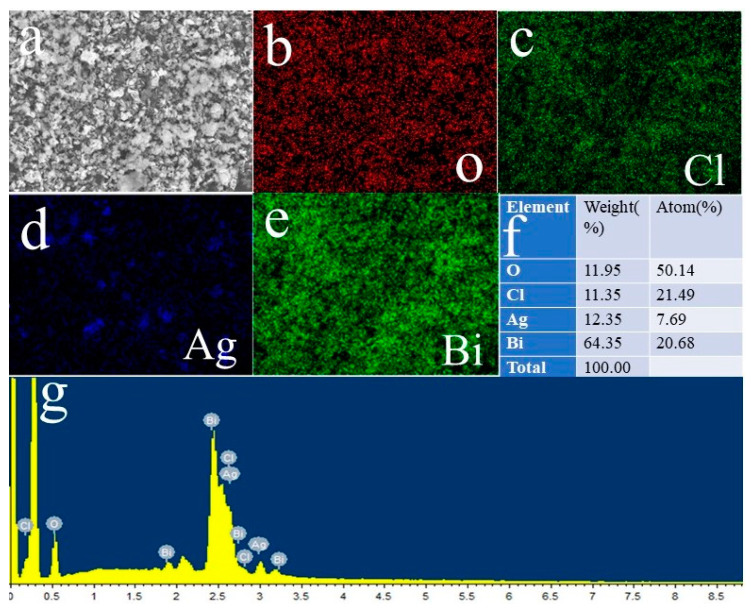
EDS elemental mapping of ABC-30 heterojunction (**a**–**g**).

The ABC-30 heterojunctions were further investigated via TEM and HRTEM. In Figure 3a, flower-like AgBiO_3_ was deposited on the surface of sheet-like BiOCl. As revealed in Figure 3b, lattice spacing of 0.34 nm and 0.31 nm was detected, corresponding to the (101) plane of BiOCl and the (104) plane of AgBiO_3_, respectively, which further proved the successful construction of the AgBiO_3_/BiOCl heterojunction. In Figure 4, the four elements Bi, O, Cl, and Ag were distributed on the heterojunction’s surface, which once again proved the formation of the AgBiO_3_/BiOCl heterojunction.

### 3.2. Photocatalytic Activity

**Figure 5:**
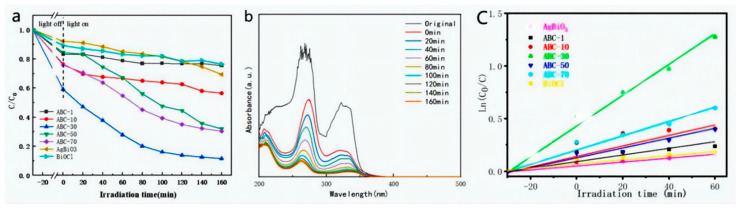
(**a**) Degradation efficiencies, (**b**) time-dependent absorption spectra of CIP, and (**c**) kinetic curves of CIP using the as-prepared photocatalysts.

Ciprofloxacin was selected to estimate the photocatalytic activity of the as-synthesized catalysts. As shown in Figure 5a, the CIP concentration possessed no significant change in the absence of photocatalysts under visible-light irradiation, suggesting a high stability. The degradation efficiency of CIP towards pure AgBiO_3_ and BiOCl was only 31% and 24% within 160 min under visible-light irradiation, indicating lower photocatalytic activity. However, with the introduction of AgBiO_3_, the photocatalytic activities of the AgBiO_3_/BiOCl heterojunction first increased and then decreased. Among the AgBiO_3_/BiOCl heterojunction, the ABC-30 heterojunction showed the optimum photocatalytic performance towards CIP, reaching 88%. This manifested that the proper addition of AgBiO_3_ could effectually enhance the photocatalytic activity, due to the enhanced light absorption and the elevated separation of photo-generated carriers. However, excessive AgBiO_3_ could cause a decrease in the degradation efficiency, attributed to the increased hindrance of interface contact, affecting the migration of photo-generated electrons [31,32]. Figure 5b revealed the UV–vis spectrum of CIP as time changed. Simultaneously, an evident decrease at 273 nm was detected. Due to the low concentration of CIP and minimal interference during the initial stage, the photocatalytic reaction should follow the pseudo-first-order kinetics model [33]: ln (*C_o_*/*C_t_*) = *kt*. As shown in Figure 5c, the reaction rate constants (*k*) of ABC-1, ABC-10, ABC-30, ABC-50, ABC-70, AgBiO_3_, and BiOCl were 0.0024 min^−1^, 0.0044 min^−1^, 0.0137 min^−1^, 0.0042 min^−1^, 0.0063 min^−1^, 0.0017 min^−1^, and 0.0019 min^−1^, respectively. The *k* of the ABC-30 heterojunction was 8.06- and 7.21-folds higher than that of the pure AgBiO_3_ and BiOCl, respectively.

**Figure 6:**
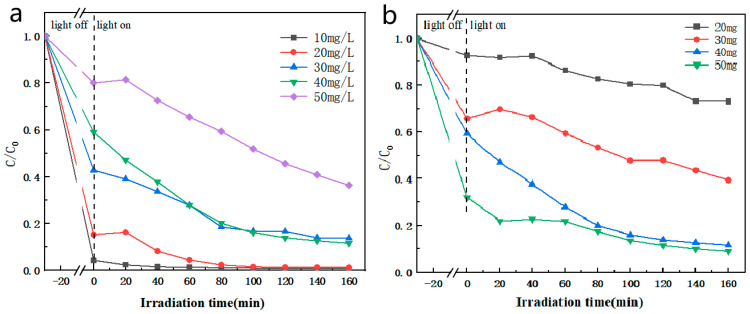
Efficiencies of (**a**) different concentrations of CIP ([ABC-30] = 1 g/L), (**b**) different amounts of ABC-30 heterojunction ([CIP] = 40 mg/L) under visible-light irradiation.

The influence of multiple reaction conditions was explored during CIP degradation. As shown in Figure 6a, the degradation effect of the different initial concentrations of CIP was investigated. When the initial concentrations of CIP were 10 mg/L, 20 mg/L, 30 mg/L, 40 mg/L, and 50 mg/L, the photocatalytic degradation rates of CIP within 160 min under visible-light irradiation were 99%, 98%, 89%, 88%, and 64%, respectively. It could be seen that the lower the initial concentration of CIP, the higher the photocatalytic degradation rate. This might be because a high concentration of CIP will quickly consume the limited active species produced by ABC-30. Therefore, the optimal initial concentration of CIP was determined to be 40 mg/L in this work. In Figure 6b, as the addition of the ABC-30 heterojunction increased from 20 mg to 40 mg, the photocatalytic degradation efficiency increased from 28% to 88% within 160 min. This indicated that the increase in photocatalysts was beneficial for photocatalytic reactions, mainly because the increase in photocatalysts could produce more active species. When the amount of photocatalysis further increased to 50 mg, there was no evident ascent in the degradation efficiency of CIP. Therefore, considering the cost of photocatalysts, the optimal amount of 40 mg was used for subsequent experimental research.

**Figure 7:**
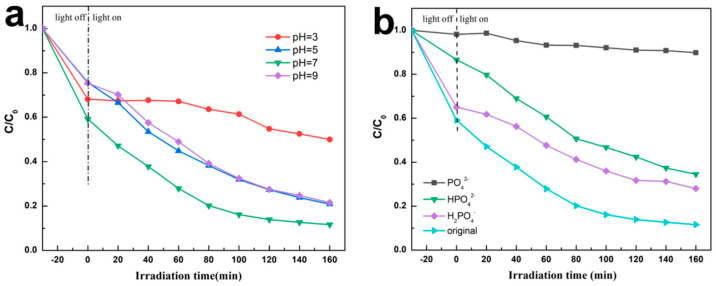
Efficiencies of CIP (**a**) at different pH, (**b**) with different inorganic salts.

Figure 7a shows the effect of the pH on the photocatalytic activity of the prepared material. With the decrease in the pH, the photocatalytic degradation activity of ABC-30 towards CIP gradually decreases. On the basis of the protonation and deprotonation processes, the surface charge of CIP largely depended on the pH of the solution. When the pH was below 6.09, the CIP surface carried a positive charge. When the pH was between 6.09 and 8.62, the surface of CIP was neutral [31]. Therefore, a decrease in the pH will cause electrostatic repulsion between positively charged photocatalyst surfaces and protonated CIP molecules, attenuating the adsorption capacity of heterostructures towards CIP and leading to a decrease in the photocatalytic degradation rate. As the pH increased to 9, the degradation efficiencies of CIP significantly abated, which might result in chemical changes in the ABC-30 heterojunction under alkaline conditions.

Actually, various inorganic ions were present in the water. So as to investigate the influence of inorganic ions towards the photocatalysts, a photo-degradation experiment with impurity anions (PO_4_^3−^, HPO_4_^2−^ and H_2_PO_4_^−^, 1 mmol) on the degradation of CIP by ABC-30 was performed. In Figure 7b, the introduction of ions had varying degrees of impact on the photocatalytic degradation of CIP. Among them, PO_4_^3−^ had the most significant inhibition on the photocatalytic process, with a removal rate of 10%. When HPO_4_^2−^ was present, the photocatalytic degradation efficiency decreased to 65%. In addition, when the H_2_PO_4_^−^ was mixed into the reaction, the photocatalytic rate also slightly decreased. Compared with HPO_4_^2−^ and H_2_PO_4_^−^, the photocatalytic efficiencies of CIP were significantly reduced with PO_4_^3−^. This might be due to PO_4_^3−^ could capture the photo-generated holes, leading to restricting the photocatalytic process. PO_4_^3−^ could be changed to HPO_4_^2−^ after capturing the photo−generated holes. Ulteriorly, HPO_4_^2−^ would be changed into H_2_PO_4_^−^ after capturing the photo−induced holes. However, the transformation of PO_4_^3−^ into HPO_4_^2−^ was more effortless than the conversion of HPO_4_^2−^ into H_2_PO_4_^−^ [34]. Consequently, the photocatalytic degradation process was obviously inhibited in the presence of PO_4_^3−^.

**Figure 8:**
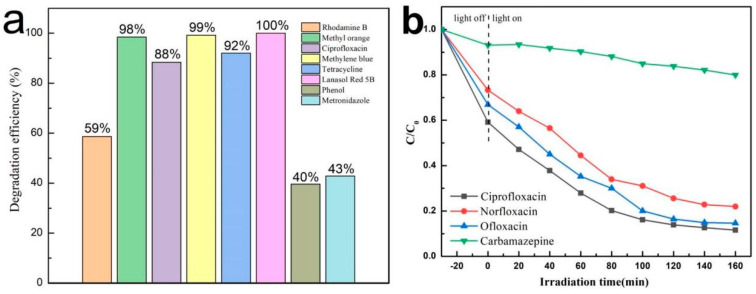
(**a**) Degradation efficiency of various pollutants in the presence of ABC-30; (**b**) photo-degradation of ciprofloxacin, ofloxacin, norfloxacin, and carbamazepine in the presence of ABC-30.

In order to investigate the practicality of the as-synthetized catalyst for environmental pollution, photocatalytic degradation experiments were conducted on rhodamine B, methyl orange, ciprofloxacin, methylene blue, tetracycline hydrochloride, Lanasol 5B, phenol, and metronidazole. In Figure 8a, the photo-degradation efficiencies of the ABC-30 heterojunction for rhodamine B, methyl orange, ciprofloxacin, methylene blue, tetracycline hydrochloride, Lannasol 5B, phenol, and metronidazole were 59%, 98%, 88%, 99%, 92%, 100%, 40%, and 43%, respectively. Obviously, it was easier for the ABC-30 heterojunction to degrade large-molecule organic dyes and more difficult to degrade small-molecule organic compounds under the same conditions. The root cause of the phenomenon could be ascribed to variable adsorption characteristics. In addition, antibiotics such as norfloxacin, ofloxacin, and carbamazepine were also degraded (Figure 8b). The degradation efficiencies of the ABC-30 heterojunction for ciprofloxacin, norfloxacin, ofloxacin, and carbamazepine were 88%, 78%, 85%, and 20%, respectively. The different photocatalytic activities might be attributed to the different molecular structures of the various organic contaminants.

**Figure 9:**
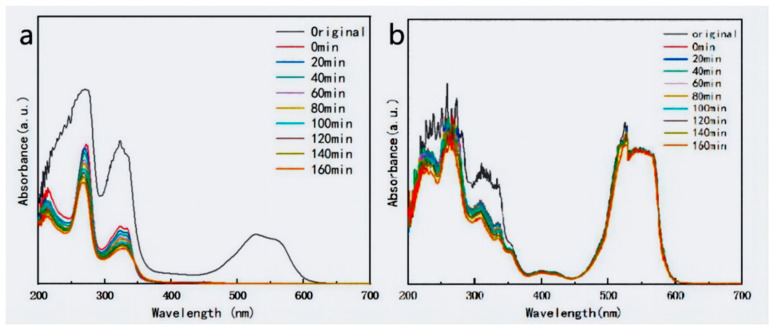
The absorption spectrum during the synergistic degradation of (**a**) CIP-LR5B, (**b**) CIP-RhB.

To further investigate the degradation performance of the as-prepared photocatalysts on organic pollutants, a mixed experiment was executed to investigate the influence of different pollutants coexisting on the degradation process. LR5B and RhB were selected to be mixed with CIP, and the degradation efficiencies are revealed in Figure 9a,b. When CIP coexisted with LR5B and RhB, respectively, the degradation efficiency of the catalyst revealed a significant decrease for CIP, possibly due to the competition between the two organic molecules for active species [15].

**Figure 10:**
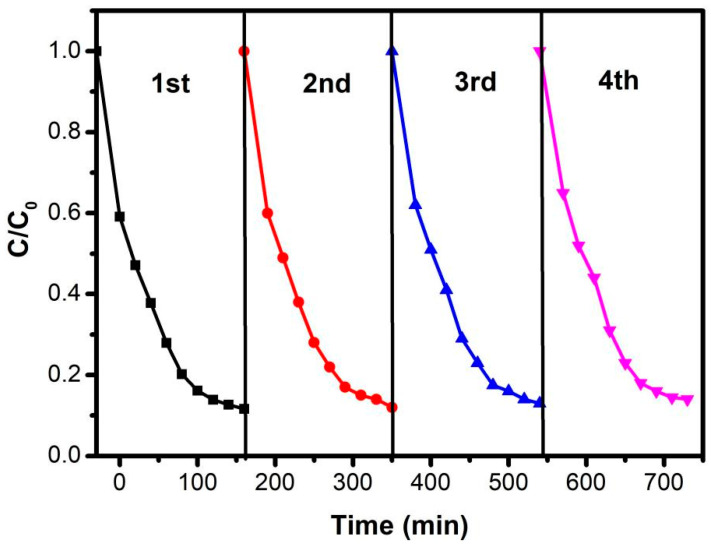
Cycle runs of ABC-30 for the degradation of CIP under visible-light irradiation.

The stability and repeatability of the ABC-30 for CIP degradation was essential. Recycle experiments were performed to investigate the stability of ABC-30, and the results are shown in Figure 10. The photocatalytic efficiencies of CIP decreased from 88% to 86% after four cycles, which suggested that the ABC-30 had excellent stability.

### 3.3. Photocatalytic Mechanism

**Figure 11:**
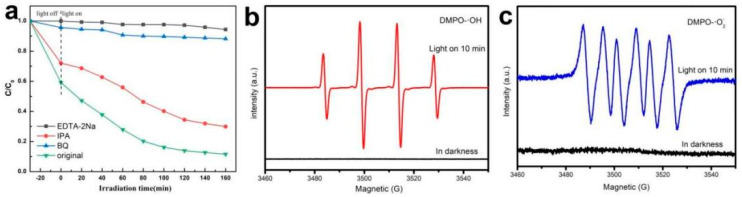
(**a**) Trapping experiments of active species during photo-degradation of CIP in the presence of ABC-30 heterojunction. (**b**) DMPO-•OH and (**c**) DMPO-•O_2_^−^ adducts on ABC-30 heterojunction.

So as to study the photocatalytic mechanism of ABC-30 heterojunction, isopropanol (IPA), disodium ethylenediaminetetraacetate (EDTA-2Na), and benzoquinone (BQ) were added to capture hydroxyl radicals (•OH), holes (h^+^), and superoxide radicals (•O_2_^−^), respectively [35]. The experimental results are shown in Figure 11a. When BQ and EDTA-2Na were, respectively, added to the reaction, the degradation efficiencies of CIP dropped sharply to 10% and 6%. The degradation processes were significantly inhibited, indicating that BQ and EDTA-2Na could effectively capture the •O_2_^−^ and h^+^ generated during the photocatalytic process. When IPA was added, the photocatalytic degradation efficiency slightly decreased. In brief, the results suggested that •O_2_^−^ and h^+^ were the main active species in the photocatalytic reactions, and •OH played an auxiliary role in degradation. In addition, ESR analysis of ABC-30 was conducted to detect •O_2_^−^ and •OH during the photo-degradation process. As described in Figure 11b,c, no characteristic signal could be investigated for both DMPO-•OH and DMPO-•O_2_^−^ in the dark. However, the signals of DMPO-•OH and DMPO-•O_2_^−^ were both detected after 10 min, which indicated that •OH and •O_2_^−^ radicals were produced in the photo-degradation processes.

**Figure 12:**
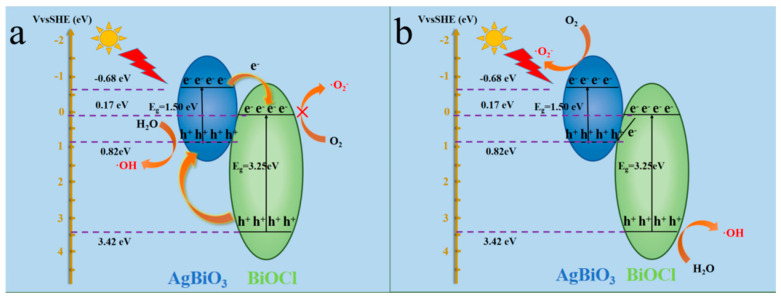
Proposed charge transfer and photocatalytic mechanisms for removal of CIP over the ABC-30 heterojunction.

According to the foregoing experimental results, a possible photocatalytic mechanism of the ABC-30 heterojunction could be supposed. In Figure 12a, the AgBiO_3_ and BiOCl surfaces could generate photo-generated electron–hole pairs under visible-light irradiation. Attributed to both the lower CB and VB potentials of BiOCl than AgBiO_3_, the photo-generated electrons of AgBiO_3_ could transfer to the CB of BiOCl. Meanwhile, the photo-generated holes of BiOCl could transfer to the VB of AgBiO_3_. Unfortunately, the CB position of BiOCl was +0.17 eV, which was more positive than the standard redox potential of E (O_2_/•O_2_^−^) (−0.33 eV vs. NHE) [30,36]. Consequently, the photo-generated electrons in the CB of BiOCl could not react with O_2_ to produce •O_2_^−^, which was not in agreement with the capture experiment. Obviously, the trapping experiment and ESR analysis had proved that •O_2_^−^, h^+^, and •OH contributed to the photo-degradation processes. Thus, the Z-scheme transfer mechanism of the ABC-30 heterojunction was proposed. In Figure 12b, the CB position of AgBiO_3_ was −0.68 eV, which was more negative than the standard redox potential of E (O_2_/•O_2_^−^) (−0.33 eV vs. NHE). Therefore, the photo-induced electrons on the conduction band of AgBiO_3_ could reduce O_2_ to •O_2_^−^. Additionally, the VB position of BiOCl was +3.42 eV [30], which was more positive than the standard redox potential of E (•OH/H_2_O) (+2.72 eV vs. NHE), suggesting that H_2_O could be oxidized to generate •OH. The Z-scheme heterojunction could effectively improve the separation efficiency of the photo-generated carriers and expand the light response range, which help to enhance the performance of the AgBiO_3_/BiOCl heterojunction. Simultaneously, the Z-scheme heterojunction could retain photo-generated electrons with high reducing ability and photo-generated holes with high oxidizing ability, thereby enhancing the photocatalytic activity, which was crucial for improving the performance of the AgBiO_3_/BiOCl heterojunction.

## 4. Conclusions

To sum up, a Z-scheme AgBiO_3_/BiOCl heterojunction with oxygen vacancies was synthetized by the water bath method. The ABC-30 heterojunction had the best removal efficiency (88%) on CIP after 160 min, which was 2.89 and 3.76 times higher than pure AgBiO_3_ and BiOCl, respectively. The photocatalytic experimental results revealed that the lower the CIP concentration, the higher the catalyst dosage, the more favorable it is for CIP removal, while the presence of PO_4_^3−^ ions or other pollutants would inhibit the removal efficiency. The experiment further investigated the degradation efficiency on pollutants such as metronidazole (43%), methyl orange (98%), tetracycline hydrochloride (92%), Lanasol 5B (100%), rhodamine B (59%), phenol (40%), norfloxacin (78%), methylene blue (99%), ofloxacin (85%), and carbamazepine (20%), which showed that the ABC-30 had good removal effects on most pollutants and possessed a certain broad-spectrum. The trapping experiment indicated •O_2_^−^ and h^+^ were the main active species for CIP degradation.

## Data Availability

The original contributions presented in the study are included in the article, further inquiries can be directed to the corresponding authors.
